# Impact of continuity of care on risk for major osteoporotic fracture in patients with new onset rheumatoid arthritis

**DOI:** 10.1038/s41598-022-14368-7

**Published:** 2022-06-17

**Authors:** Seung Hoon Kim, Hyunkyu Kim, Sung Hoon Jeong, Suk-Yong Jang, Eun-Cheol Park

**Affiliations:** 1 Department of Preventive Medicine, Yonsei University College of Medicine, 50-1 Yonsei-ro, Seodaemun-gu, Seoul, 03722 Republic of Korea; 2 Institute of Health Services Research, Yonsei University, Seoul, Republic of Korea; 3 Department of Public Health, Graduate School, Yonsei University, Seoul, Republic of Korea; 4 Department of Psychiatry, Yonsei University College of Medicine, Seoul, Republic of Korea; 5 Department of Healthcare Management, Graduate School of Public Health, Yonsei University, Seoul, Republic of Korea

**Keywords:** Disease prevention, Health policy, Public health, Rheumatology

## Abstract

There is a clear relationship between rheumatoid arthritis (RA) and major osteoporotic fracture (MOF), although there is limited evidence on the effect of continuity of care (COC) on MOF in these patients. We investigated the association between COC and risk of MOF, including fractures of the lumbar spine and pelvis, forearm, and hip, among newly diagnosed RA patients aged ≥ 60 years. A total of 8715 incident RA patients from 2004 to 2010 were included from the Korean National Health Insurance Service-Senior cohort database. Participants were categorized into a good and bad COC group according to the COC index. The cumulative incidence of MOF was higher in RA patients with bad than in those with good COC (*p* < 0.001). The incidence rates of MOF were 4439 and 3275 cases per 100,000 person-years in patients with bad and good COC, respectively. RA patients with bad COC had an increased incidence of overall MOF (adjusted hazard ratio, 1.32; 95% confidence interval, 1.14–1.53), with the highest increase in risk being that of forearm fracture. An increased MOF risk in patients with bad COC was predominantly observed in females. This study suggested that interventions that can improve COC in patients with RA should be considered.

## Introduction

Rheumatoid arthritis (RA) is a progressive inflammatory disease characterized by chronic symmetrical polyarticular and extra-articular involvement^1^. Patients with RA may develop skeletal complications, including generalized bone loss, osteopenia, and osteoporotic fracture^2,3^. Among the comorbidities, major osteoporotic fracture (MOF) not only reduces the quality of life in the elderly population but also increases hospitalization and immediate- and long-term mortality risk; it is thus clinically important to find preventable factors associated with MOF in RA patients^4,5^. RA per se is a risk factor for the development of MOF, and risk of MOF increases as the disease progresses and chronic systemic inflammation increases^6,7^. Moreover, medications for RA are also associated with fracture risk^8^. The appropriate treatment for RA patients can improve the quality of life and increase life expectancy in the elderly with RA by preventing future MOF.

Continuity of care (COC), an essential concept for high-quality patient care, is the process by which patients and providers maintain an ongoing partnership to effectively meet the patients' healthcare needs^9^. Better COC is known to improve patient outcomes and satisfaction^10,11^. However, patients with chronic diseases involving a relatively long treatment period do not receive COC and often demonstrate “doctor-shopping” behavior^12^. In RA specifically, the goal of treatment is remission rather than cure, and relapse is common when medications are arbitrarily stopped or tapered after remission^13^. Subsequently, the patient may mistakenly believe that the recurrence resulted from poor quality of care at the initial hospital and may seek care from other hospitals. There is especially no regulation due to the attributes of the medical system in the Republic of Korea in which there is no primary care physician acting as a gatekeeper; rather, a specialist oversees primary care at the clinic^14^. This can lead to many fragmented visits.

Although there are several published studies reporting that better COC can reduce comorbidities in chronic diseases, such as diabetes and hypertension, only a few studies report the effect of COC on comorbidities in RA patients^14–17^. Therefore, this study aimed to investigate the association between the continuity of ambulatory care and incidence of MOF defined as fracture of the lumbar spine and pelvis, forearm, or hip in elderly patients with RA using a nationally representative sample from the National Health Insurance Service (NHIS)-Senior cohort 2002–2013.

## Methods

### Data and sample

This study used data from the 2002–2013 NHIS–Senior cohort provided by the NHIS of the Republic of Korea. The NHIS is the sole medical insurer managed by the government in the Republic of Korea that provides a system of universal healthcare coverage to the citizens of the Republic of Korea. All citizens, except those eligible for medical aid, are obligated to enroll in the NHIS. The National Health Information Database was developed by the NHIS and contains personal information and demographic details of all those enrolled for the purpose of collecting insurance premium and subscription data for reimbursement^18^.

The NHIS–Senior cohort refers to a representative sample cohort created by randomly selecting 558,147 adults aged ≥ 60 years, comprising 10% of the total eligible population in the Republic of Korea in 2002. Participants were followed-up for a period of 12 years until 2013, unless they were disqualified due to death or emigration^19^. The database includes information on reimbursement for each medical service and includes basic patient information, diagnostic codes, expenses incurred, and death-related information.

### Study sample

Our study sample included only patients who met the following criteria: (1) newly diagnosed RA patients in 2004–2010 and (2) at least four visits to an ambulatory clinic for RA within 2 years after initial diagnosis. Incident RA patients were defined as those who were diagnosed with diseases under the M05 code of the 10th revision of the International Statistical Classification of Diseases and Related Health Problems (ICD-10) during outpatient treatment. To enumerate only new onset RA patients, those diagnosed with RA between January 1, 2002 and December 31, 2003 were excluded^20^. Finally, to secure a time interval of at least 2 years to measure COC, patients diagnosed with RA for the first time after 2011 were excluded.

### Identification of MOF

The primary outcome was defined as an incidence of MOF that met the following criteria: (1) patients diagnosed with fracture of the lumbar spine and pelvis, forearm, or hip (ICD-10 codes, S32, S52, and S72), (2) patients with a history of two or more outpatient visits or one or more hospitalizations owing to fracture after diagnosis, and (3) cases where the fracture occurred 2 years after the diagnosis of RA to consider the period for calculating the COC index.

### Measuring COC

The characteristics of the medical delivery system in the Republic of Korea, which is not limited in terms of selecting the patient's preferred primary care provider, were considered in the measurement of COC. Therefore, the COC index, in this case, refers to the consistency of care^17^. There are various methods for measuring COC, of which the one most widely used in research is classification based on whether or not a primary health-care provider is designated^21^. The COC index, proposed by Bice et al., is the most representative index and is measured by combining the two aspects of visit concentration and visit distribution. The formula for measuring the COC index is as follows:

$${\text{COC index}} = \frac{{\mathop \sum \nolimits_{j = 1}^{M} n_{j}^{2} - N}}{{N\left( {N - 1} \right)}},$$

where n_j_ was defined as the number of visits to the provider j, M is the number of medical service providers, and N is the total number of visits^22^. The COC index ranges from 0 to 1, where 0 means outpatient visits are distributed to different providers, and 1 means they are focused on one provider.

**Figure 1:**
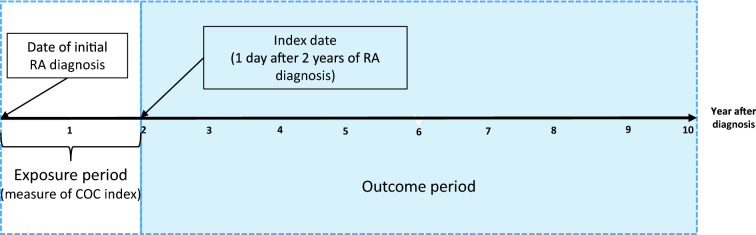
The schematic diagram of the time frame for study.

We selected a 2-year exposure period to ensure longitudinal continuity, as in the previous study^23^, and reflected the COC index of participants with four or more visits^23,24^. Therefore, the COC index within the first 2 years of RA diagnosis is determined according to all outpatient visits (Fig. 1). The reason for specifying the minimum number of visits when calculating the COC index is that the COC index is relatively easy to reach the maximum value of 1 or the minimum value of 0 with a small number of visits^24^. In this study, we used a cut-off point of 0.75 for the COC index, which has been extensively validated in previous studies^25,26^. Patients were thus classified as having good or bad COC depending on whether more or less than 75% of the outpatient visits were from the same physician during the 2 years following diagnosis of RA^27^. Since there are several methods of measuring COC, a sensitivity analysis was also performed by calculating the usual provider of care (UPC), another representative method. As in the previous study, if the UPC was 0.75 or higher, the patient was defined as having good COC^28^.

### Covariates

This study considered age (60–64, 65–69, 70–74, 75–79, 80 + years), sex (male and female), income-level quintile, residential area (urban or rural), registered disability (yes or no), Charlson Comorbidity index (CCI; 0–1, 2, 3, or  ≥ 4), history of osteoporosis (yes or no), RA severity (mild or severe), systemic glucocorticoid exposure (yes or no), and hospital-level variables as covariates. Hospital-level variables included hospital classification (general hospital, hospital, or clinic), hospital location (metropolitan, urban, or rural), number of beds (< 30, < 300, < 1000, or  ≥ 1000), and ownership (public, corporate, or private). The CCI was calculated by the weighting and scoring of comorbidity conditions using Quan’s method, with additional points given to comorbidities that affect the health outcomes of patients^29,30^. Patients were classified as having severe RA according to the prescription claim for tumor necrosis factor inhibitors (TNFis), biologics, (infliximab, adalimumab, golimumab, certolizumab pegol, or etanercept), non-TNFi biologics, (abatacept, rituximab, tocilizumab, or anakinra), or Janus kinase inhibitors (JAKis) (tofacitinib)^31–33^. Patients who had used oral glucocorticoids for ≥ 6 months were categorized into the systemic glucocorticoid exposure group^34^. The hospital-level variables were based on the health-care institution most frequently visited by the patient for outpatient treatment^17^.

### Statistical analyses

We used the Chi-square test for categorical variables to compare the distribution of baseline characteristics. Aalen-Johansen estimators were used to determine the cumulative incidence of MOF and 95% confidence interval (CI), and Gray’s k-sample test was conducted to compare the cumulative incidence^35,36^. A generalized estimating equation using a Poisson distribution was conducted to calculate the incidence rate (IR) of MOF and 95% CI. The IR was expressed as the number of MOF per 100,000 person-years. The effect size was expressed as a hazard ratio (HR) using the Cox proportional hazards model. We set time zero (index date) as 1 day after 2 years of RA diagnosis for each patient. We defined the survival time used in the survival analyses as the number of months from time zero to the date of MOF development, date of death, or December 31, 2013, whichever came first. The log transformation for the negative log of the estimated survivor function and Schoenfeld residuals were used to assess proportional hazard assumption. A Fine and Gray competing risk model with death as a competing risk was conducted along with cause-specific hazard model. Furthermore, since age may act as an important confounder in this study, an additional analysis was performed using age as a continuous variable and attained age (age as time scale)^37^. For the sensitivity analysis, the association between COC and the risk of MOF was investigated using the COC index segregated into four categories by 0.25 units and UPC. Stratified analyses according to age group, sex, and history of osteoporosis were also performed. Additionally, dependent subgroup analyzes were performed to examine whether COC had different effects depending on the MOF subtype. All calculated *p*-values were two–sided; *p*-values < 0.05 were considered significant. We performed all analyses using SAS software version 9.4 (SAS Institute, Cary, NC, USA) and R version 4.0.3 (Vienna, Austria; Rproject.org/).

### Ethics approval and consent to participate

This study adhered to the tenets of the Declaration of Helsinki and was approved by the Institutional Review Board of Severance Hospital at Yonsei University College of Medicine (IRB no. 4-2021-0984). The need of informed consent was waived by the IRB of Severance Hospital at Yonsei University College of Medicine, as data of the NHIS–Senior cohort do not contain any personally identifiable information.

## Results

**Figure 2:**
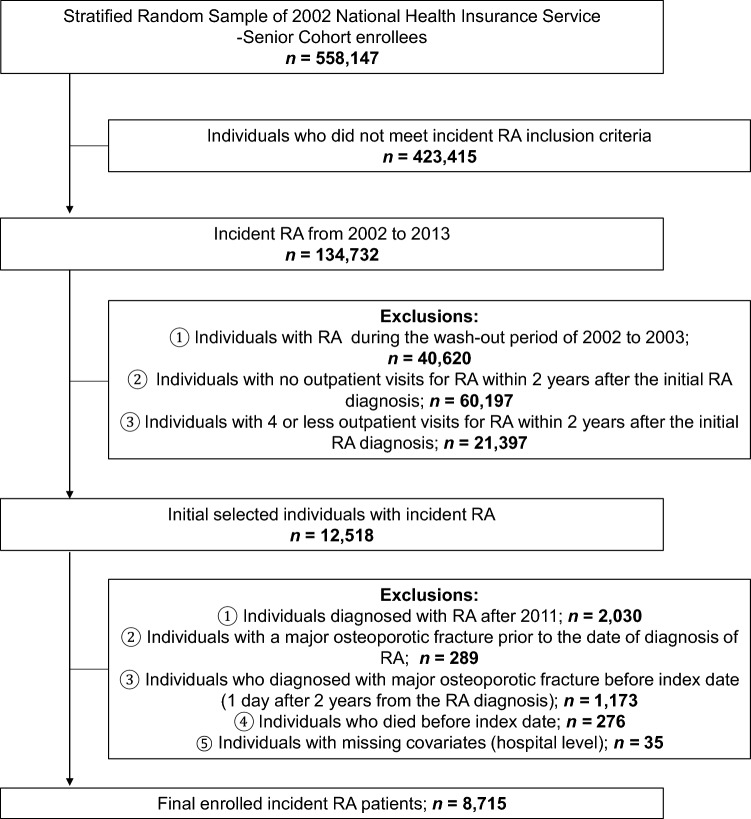
Details of study population. RA, Rheumatoid arthritis.

From January 1, 2002 to December 31, 2013, a total of 134,732 RA patients met the inclusion criteria. Among them, patients with RA were excluded during the washout period of 2002–2003 (n = 40,620). As a next step, we excluded patients who had no outpatient visits for RA within 2 years after the first RA diagnosis (n = 60,197) and patients who had fewer than four outpatient visits within 2 years (n = 21,397). Among the initially selected patients with incident RA, those diagnosed with RA after 2011(n = 2030), with MOF before the RA diagnosis (n = 289) or index date (n = 1173), incident RA patients who died before the index date (n = 276), and those with missing covariates (n = 35) were also excluded. Finally, 8715 new onset RA patients from 2004 to 2010 were included in this study (Fig. 2).

The baseline characteristics of the study population according to the COC index are described in Table 1. Of the total 8715 RA cohort, 1088 (12.5%) patients had a bad COC index. Patients with bad COC index tended to have a history of osteoporosis and be exposed to systemic glucocorticoid. The mean follow-up period was 4.4 years, and 38,228 person-years were observed. During the follow-up period, 1310 (15.0% of the RA cohort) patients with RA developed MOF.

**Table 1:** Baseline characteristics according to level of continuity of care.

Variables	Continuity of care				*p*-value
	Good (COC index ≥ 0.75)		Bad (COC index < 0.75)		
Total (n = 8715)	7627	(87.5)	1088	(12.5)	
**Sex**					0.346
Male	2116	(27.7)	287	(26.4)	
Female	5511	(72.3)	801	(73.6)	
**Age**					< 0.001
60–64	583	(7.6)	127	(11.7)	
65–69	2525	(33.1)	428	(39.3)	
70–74	2274	(29.8)	317	(29.1)	
75–79	1364	(17.9)	148	(13.6)	
80–	881	(11.6)	68	(6.3)	
**Household income level**					0.143
Low	971	(12.7)	135	(12.4)	
Medium–low	1568	(20.6)	206	(18.9)	
Medium	1402	(18.4)	177	(16.3)	
Medium–high	1336	(17.5)	207	(19.0)	
High	2350	(30.8)	363	(33.4)	
**Region**					0.202
Urban	2792	(36.6)	420	(38.6)	
Rural	4835	(63.4)	668	(61.4)	
**Registered disability**					0.253
No	7559	(99.1)	1082	(99.4)	
Yes	68	(0.9)	6	(0.6)	
**Charlson comorbidity index (CCI)**					0.044
0–1	2822	(37.0)	374	(34.4)	
2	2258	(29.6)	306	(28.1)	
3	1246	(16.3)	190	(17.5)	
4–	1301	(17.1)	218	(20.0)	
**History of osteoporosis**					< 0.001
No	5690	(74.6)	708	(65.1)	
Yes	1937	(25.4)	380	(34.9)	
**RA severity**					< 0.001
Mild	7606	(99.7)	1066	(98.0)	
Severe	21	(0.3)	22	(2.0)	
**Systemic glucocorticoid exposure**					< 0.001
No	6745	(88.4)	760	(69.9)	
Yes	882	(11.6)	328	(30.1)	
**Hospital level**					
Hospital classification					< 0.001
General hospital	1073	(14.1)	251	(23.1)	
Hospital	223	(2.9)	56	(5.1)	
Clinic	6331	(83.0)	781	(71.8)	
**Hospital location**					< 0.001
Metropolitan	1440	(18.9)	273	(25.1)	
Urban	1638	(21.5)	255	(23.4)	
Rural	4549	(59.6)	560	(51.5)	
**Number of beds**					< 0.001
< 30	5382	(70.6)	666	(61.2)	
< 300	1214	(15.9)	186	(17.1)	
< 1000	546	(7.2)	128	(11.8)	
≥ 1000	485	(6.4)	108	(9.9)	
**Ownership**					< 0.001
Public	1351	(17.7)	157	(14.4)	
Corporate	1292	(16.9)	285	(26.2)	
Private	4984	(65.3)	646	(59.4)	

Values are presented as number (%).

*COC* continuity of care.

*p*-values were obtained by Chi-square test.

We observed a significant difference in the cumulative incidence of developing MOF during the entire follow-up period between RA patients with good and bad COC indices (*p* < 0.001 for the Gray’s test, Supplementary Fig. 1). The 8-year cumulative incidence risks of MOF were 27.91% and 21.00% in RA patients with bad and good COC indices, respectively (Table 2).

**Table 2:** Cumulative incidence (%) of major osteoporotic fracture in patients with newly developed rheumatoid arthritis.

Cumulative time frame	Continuity of care			
	Good (COC index ≥ 0.75)		Bad (COC index < 0.75)	
	Cumulative incidence (%)	95% CI	Cumulative incidence (%)	95% CI
1 year	2.92	(2.56–3.32)	4.04	(2.99–5.33)
2 years	5.92	(5.40–6.47)	7.65	(6.16–9.34)
3 years	8.88	(8.23–9.55)	12.14	(10.23–14.22)
4 years	11.76	(11.00–12.54)	16.02	(13.81–18.37)
5 years	13.92	(13.08–14.78)	18.52	(16.12–21.04)
6 years	16.54	(15.59–17.53)	21.82	(19.16–24.60)
7 years	18.88	(17.77–20.01)	25.87	(22.68–29.17)
8 years	21.00	(19.39–22.66)	27.91	(23.95–31.99)

Cumulative incidence was estimated using the Aalen-Johansen estimator.

*COC* continuity of care, *CI* confidence interval.

During the entire follow-up period, 222 new MOF cases were identified from 1088 RA patients with a bad COC index (Table 3). After adjusting for all covariates, RA patients with a bad COC index were 1.32 times more likely to develop MOF than those with a good COC index (adjusted HR, 1.32; 95% CI, 1.14–1.53; Table 3). When the age was adjusted as a continuous variable and the cox model with attained age was conducted, the results were similar to the main results (Table 3). The results using the Fine and Gray subdistribution hazard models (adjusted HR, 1.32; 95% CI, 1.13–1.54) and the cause-specific model were similar. Unadjusted and confounder-adjusted estimates are presented in Supplementary Table 1.

**Table 3:** Continuity of care and major osteoporotic fracture risk in patients with newly developed rheumatoid arthritis.

	Major osteoporotic fracture	
	Good COC index (COC index ≥ 0.75)	Bad COC index (COC index < 0.75)
n	7627	1088
No. of fracture	1088	222
Person-years	33,226	5002
Incidence rate (95% CI)^a^	3275 (3086–3475)	4439 (3891–5063)
**Adjusted hazard ratio (95% CI**)^b^		
Model 1 (age as a categorical variable)	1.00 (Reference)	1.32 (1.14–1.53)
Model 2 (age as a continuous variable)	1.00 (Reference)	1.29 (1.11–1.46)
Model 3 (Cox model with attained age)	1.00 (Reference)	1.31 (1.12–1.51)

Major osteoporotic fracture included fracture of lumbar spine and pelvis, forearm, or hip.

*COC* continuity of care, *CI* confidence interval.

^a^Events per 100,000 person-years.

^b^Adjusted with all covariates shown in Table 1.

**Figure 3:**
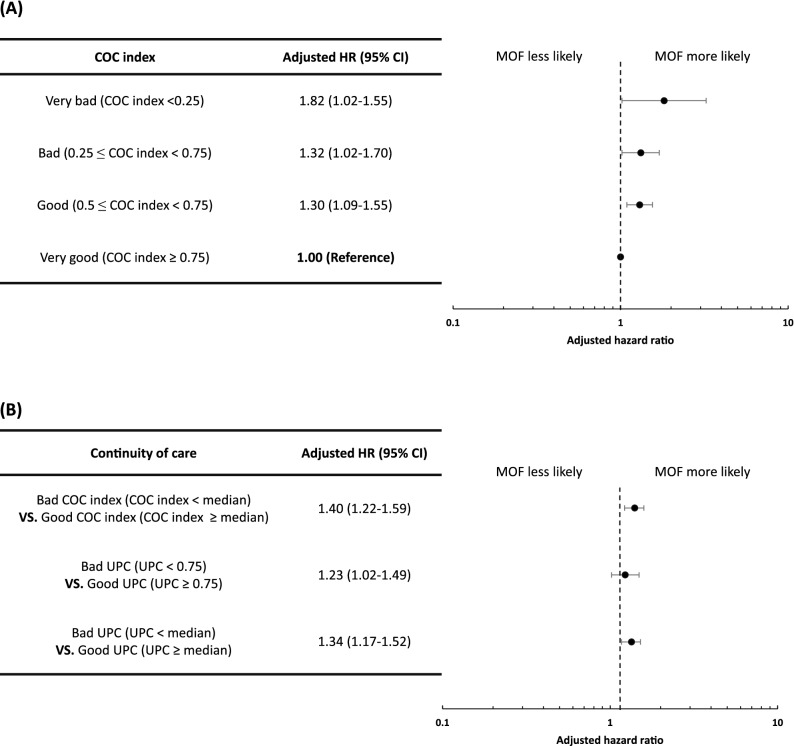
Sensitivity analyses using several measurements of continuity of care. (**A**) Analysis of the association between continuity of care (COC) and risk of major osteoporotic fracture (MOF) by classifying the COC index into four categories. (**B**) Analysis of association between COC and risk of MOF by different cut-offs in COC index and usual provider of care (UPC). UPC = N_*u*_/N; where N is the total number of outpatient visits and N_*u*_ is the number of visits to routine health care providers. *COC* continuity of care, *MOF* major osteoporotic fracture, *UPC* usual provider of care.

In the sensitivity analyses, when the COC index was divided into four groups, the risk of MOF was highest when the COC index was the worst (adjusted HR, 1.82; 95% CI, 1.02–3.24; Fig. 3). Similar results were obtained when measurements were made using the UPC rather than the COC index (adjusted HR, 1.23; 95% CI, 1.02–1.49; Fig. 3).

Table 4 shows the association of the COC index with the risk of MOF subtype and risk of hospitalization for MOF treatment in RA patients. After adjusting for all covariates, the risk of fracture of the lumbar spine and pelvis increased by 1.33 times (adjusted HR, 1.33; 95% CI, 1.09–1.62) and the risk of fracture of the forearm by 1.49 times (adjusted HR, 1.49; 95% CI, 1.11–1.99) in RA patients with a bad COC index compared with those with a good COC index. RA patients with a bad COC index were 1.29 times more likely to be hospitalized for MOF treatment (adjusted HR, 1.29; 95% CI, 1.07–1.56).

**Table 4:** Hazards ratio by osteoporotic fracture type and hospitalisation risk, according to continuity of care type.

Variables	Number of subjects	Number of fractures	Person years	Incidence rate (95% CI) per 100,000 person years	Adjusted hazard ratio^a^ (95% CI)
**Fracture of lumbar spine and pelvis**					
Good COC index (COC index ≥ 0.75)	7627	577	33,226	1737 (1601–1884)	**1.00 (Reference)**
Bad COC index (COC index < 0.75)	1088	123	5002	2459 (2061–2935)	1.33 (1.09–1.62)
**Fracture of forearm**					
Good COC index (COC index ≥ 0.75)	7627	259	33226	780 (690–880)	**1.00 (Reference)**
Bad COC index (COC index < 0.75)	1088	56	5002	1200 (862–1455)	1.49 (1.11–1.99)
**Fracture of hip**					
Good COC index (COC index ≥ 0.75)	7627	252	33,226	758 (670–858)	**1.00 (Reference)**
Bad COC index (COC index < 0.75)	1088	43	5002	860 (638–1159)	1.13 (0.81–1.58)
**Hospitalisation for fracture treatment**					
Good COC index (COC index ≥ 0.75)	7627	671	33,226	2020 (1872–2178)	**1.00 (Reference)**
Bad COC index (COC index < 0.75)	1088	134	5002	2679 (2262–3173)	1.29 (1.07- 1.56)

*COC* continuity of care, *CI* confidence interval.

^a^Adjusted with all covariates.

Table 5 presents the association between the COC index and risk of MOF according to sex, age, and history of osteoporosis. The subsequent risk of fracture in RA patients significantly increased in female patients with a bad COC index (adjusted HR, 1.33; 95% CI, 1.14–1.62), and in patients in their 60 s (adjusted HR, 1.53; 95% CI, 1.23–1.90). Furthermore, a bad COC index increased the risk of fracture regardless of a history of osteoporosis.

**Table 5:** Continuity of care and major osteoporotic fracture risk stratified by sex, age, and osteoporosis history.

Variables	Number of subjects	Number of fractures	Person years	Incidence rate (95% CI) per 100,000 person years	Adjusted hazard ratio (95% CI)
**Sex**					
Male					
Good COC index (COC index ≥ 0.75)	2116	152	8770	1733 (1479–2032)	**1.00 (Reference)**
Bad COC index (COC index < 0.75)	287	28	1240	2259 (1560–3271)	1.23 (0.81–1.88)
Female					
Good COC index (COC index ≥ 0.75)	5511	936	24,457	3827 (3590–4080)	**1.00 (Reference)**
Bad COC index (COC index < 0.75)	801	194	3762	5157(4480–5936)	1.33 (1.14–1.62)
**Age**					
60–69 years					
Good COC index (COC index ≥ 0.75)	3108	378	15,458	2445 (2211–2705)	**1.00 (Reference)**
Bad COC index (COC index < 0.75)	555	113	2798	4038 (3358–4856)	1.53(1.23–1.90)
70 years or over					
Good COC index (COC index ≥ 0.75)	4519	710	17768	3996 (3713–4301)	**1.00 (Reference)**
Bad COC index (COC index < 0.75)	533	109	2204	4947 (4100–5968)	1.13 (0.92–1.39)
**History of osteoporosis**					
No					
Good COC index (COC index ≥ 0.75)	5690	713	25,362	2811 (2612–3025)	**1.00 (Reference)**
Bad COC index (COC index < 0.75)	708	124	3322	3733 (3130–4451)	1.39 (1.14–1.69)
Yes					
Good COC index (COC index ≥ 0.75)	1937	375	7864	4768 (4309–5276)	**1.00 (Reference)**
Bad COC index (COC index < 0.75)	380	98	1680	5834 (4786–7112)	1.38 (1.10–1.74)

Adjusted with all covariates.

*COC* continuity of care, *CI* confidence interval.

## Discussion

In this study, we examined the association between COC and the risk of MOF in patients with RA. RA patients with a bad COC had a 32% greater risk of MOF after adjusting for all covariates. The cumulative incidence of MOF was 6.91% greater for patients with a bad COC than for those with a good COC index.

The association between RA and MOF is higher with chronic systemic inflammation, decreased physical activity, and vitamin D deficiency^7^. A recent study reported that drugs used for RA, including disease-modifying antirheumatic drugs, in addition to glucocorticoids, which are classically known as risk factors for fracture, are associated with fracture risk^8^. Our study suggests that better COC may reduce the risk of MOF in elderly RA patients. However, the underlying mechanism was not elucidated. One possible hypothesis is that COC may benefit patients by enhancing their knowledge about the disease, motivating them to adhere to their physician's advice^38^. The increase in compliance according to COC may have a positive effect on the disease course, such as reducing chronic inflammation.

Results of the stratified analyses showed an increased risk of MOF with a bad COC index in female RA patients. Both genetic and hormonal factors influence sex differences in the association between a bad COC index and the risk of MOF. Female RA patients have higher disease activity scores and more severe dysfunction, which may have influenced this association^39,40^. Furthermore, a bad COC index was associated with subsequent risk of MOF in RA patients in the younger sector of the study population. It is well known that the prevalence of associated systemic symptoms, disease progression, and functional outcomes may vary depending on the age of onset of RA^41^. It is assumed that distinct characteristics of laboratory findings or phenotypes in late-onset RA, which are different from those of younger-onset RA^42^, affect RA and the subsequent risk of MOF, although additional studies should be considered.

A bad COC index among RA patients did not increase the risk of hip fracture, although the risk of lumbar spine and pelvis, and forearm fractures increased significantly, concurring with previous studies that risk factors for fractures vary depending on the skeletal site^43^. Particularly, the differences between the risk factors for the distal radius and those for hip fracture may be the cause of this^44,45^.

The paradigm of recognition and treatment of RA has changed over the past two decades, and remission and damage prevention have become the major treatment goals^46^. The prioritization of evaluation of suspected inflammatory arthritis within a few weeks of onset, frequent re-evaluation to achieve objective determination of remission, and aggressive adjustment of treatment are widely accepted as the standard of care and feature as major treatment guidelines^31,32^.

To support these principles and to achieve these standards in practice, COC is important in patients with RA. The government in the Republic of Korea introduced the Chronic Disease Care System (CDCS) for patients with hypertension and diabetes in 2012 to improve the quality of treatment and to contain costs. Participation of patients in the CDCS program assures a reduction of outpatient out-of-pocket costs from the usual 20 to 30% of the total cost; there is also the provision for health support services, such as education, if hypertension or diabetes patients choose their preferred primary clinic and continue to receive treatment at the same institution^47^. COC can be improved in diabetic patients with the implementation of the CDCS pilot project^47^. Our results suggest that chronic disease management initiatives to improve COC may not be limited to patients with hypertension and diabetes but could be extended to RA patients as well.

Our study has some limitations. The first is the accuracy in diagnosing MOF. However, in defining patients with MOF, we tried to overcome the limitation of claim data by including only those patients who visited the outpatient clinic twice or more or were hospitalized at least once. Second, unhealthy behaviors such as smoking and alcohol consumption may also affect the risk of MOF; however, these could not be confirmed in the absence of relevant information in the original dataset. Third, the prescriptions of biologics, including TNFis or non-TNFi biologics, were used to adjust the severity of RA. However, the severity could not be fully reflected, as the laboratory tests of each RA patient were not confirmed due to the nature of the claim data. Lastly, causality cannot be inferred owing to the observational nature of the study, even though we adjusted for possible confounders. There might also be reverse causality in RA patients with physical limitation due to MOF that lead to less visits of clinic despite excluding patients diagnosed with MOF prior to the onset of RA. Future well-designed prospective studies are warranted to confirm the causality between COC and risk of MOF in RA patients.

Despite these limitations, this study has several notable strengths. First, to the best of our knowledge, this is the first study to investigate the association between COC and the risk of MOF. Subsequently, due to the characteristics of Korea’s NHIS, the NHIS–Senior cohort, which is based on claim data, does not target patients only in a specific hospital, institution, or area, but rather represents the entire population over 60 years of age in the Republic of Korea. Thus, this study was conducted on a representative population of RA patients aged ≥ 60 years in the Republic of Korea. Furthermore, as the NHIS–Senior cohort used in this study is a large sample from a 12-year period with a relatively small number of follow-up losses, the association between COC and the risk of MOF could be observed for a sufficient duration.

## Conclusions

In conclusion, senior RA patients with a bad COC index had an increased risk of developing MOF compared with those with a good COC index. Among MOF subtypes, a bad COC index was associated with increased subsequent risk of fracture of the lumbar spine, pelvis, and forearm. The association between COC and the subsequent risk of developing MOF in RA patients was prominent in female patients. Due to the nature of observational studies, causality cannot be inferred, but it is necessary to educate RA patients that COC is a good way to improve disease progression in clinical practice. Moreover, policymakers should consider to adopt policies to improve COC in these patients, as well as in patients with chronic diseases such as hypertension and diabetes.

## Supplementary Information

Supplementary Information.

## Data Availability

No data are available as the National Health Information Database is accessible only by researchers authorized by the National Health Insurance Service.

## Abbreviations

CCI: Charlson comorbidity index
CDCS: Chronic disease care system
CI: Confidence interval
COC: Continuity of care
HR: Hazard ratio
ICD-10: 10th revision of the International Statistical Classification of Diseases and Related Health Problems
IR: Incidence rate
JAKi: Janus kinase inhibitor
MOF: Major osteoporotic fracture
NHIS: National Health Insurance Service
RA: Rheumatoid arthritis
TNFi: Tumor necrosis factor inhibitor
UPC: Usual provider of care
