# Supplementary material for: Impact of continuity of care on risk for major osteoporotic fracture in patients with new onset rheumatoid arthritis
Source: Sci Rep. 2022 Jun 17;12:10189. doi: 10.1038/s41598-022-14368-7 (PMC9205920; doi:10.1038/s41598-022-14368-7)

Supplementary Information

SH Kim, H Kim, SH Jeong, et al. Impact of continuity of care on risk for major osteoporotic fracture in patients with new onset rheumatoid arthritis

Supplementary Table 1. Results of unadjusted and confounder-adjusted association between continuity of care and the risk of major osteoporotic fracture in patients with newly developed rheumatoid arthritis

Supplementary Figure 1. Major osteoporotic fracture cumulative incidence in patients with good and bad continuity of care index. p-value for Gray's test < 0.001.

Supplementary Table 1. Results of unadjusted and confounder-adjusted association between continuity of care and the risk of major osteoporotic fracture [a] in patients with newly developed rheumatoid arthritis

Variables	Crude		Model 1 [b]		Model 2 [c]		Model 3 [d]	
	HR	95 % CI	HR	95 % CI	HR	95 % CI	HR	95 % CI
**Continuity of care**								
Good (COC index ≥ 0.75)	**1.00 (Reference)**		**1.00 (Reference)**		**1.00 (Reference)**		**1.00 (Reference)**	
Bad (COC index< 0.75)	1.35	(1.17 – 1.56)	1.42	(1.23 – 1.64)	1.41	(1.22 – 1.63)	1.32	(1.14 – 1.53)

[a] Fracture of spine, pelvis, forearm, or hip.

[b] Adjusted with age, sex, household income level, and region.

[c] Adjusted with age, sex, household income level, region, and hospital level (classification, location, ownership, number of beds).

[d] Adjusted with all variables including health-related factors.

Abbreviations: COC, continuity of care; CI, confidence interval.

Supplementary Figure 1. Major osteoporotic fracture cumulative incidence in patients with good and bad continuity of care index. p-value for Gray's test < 0.001.

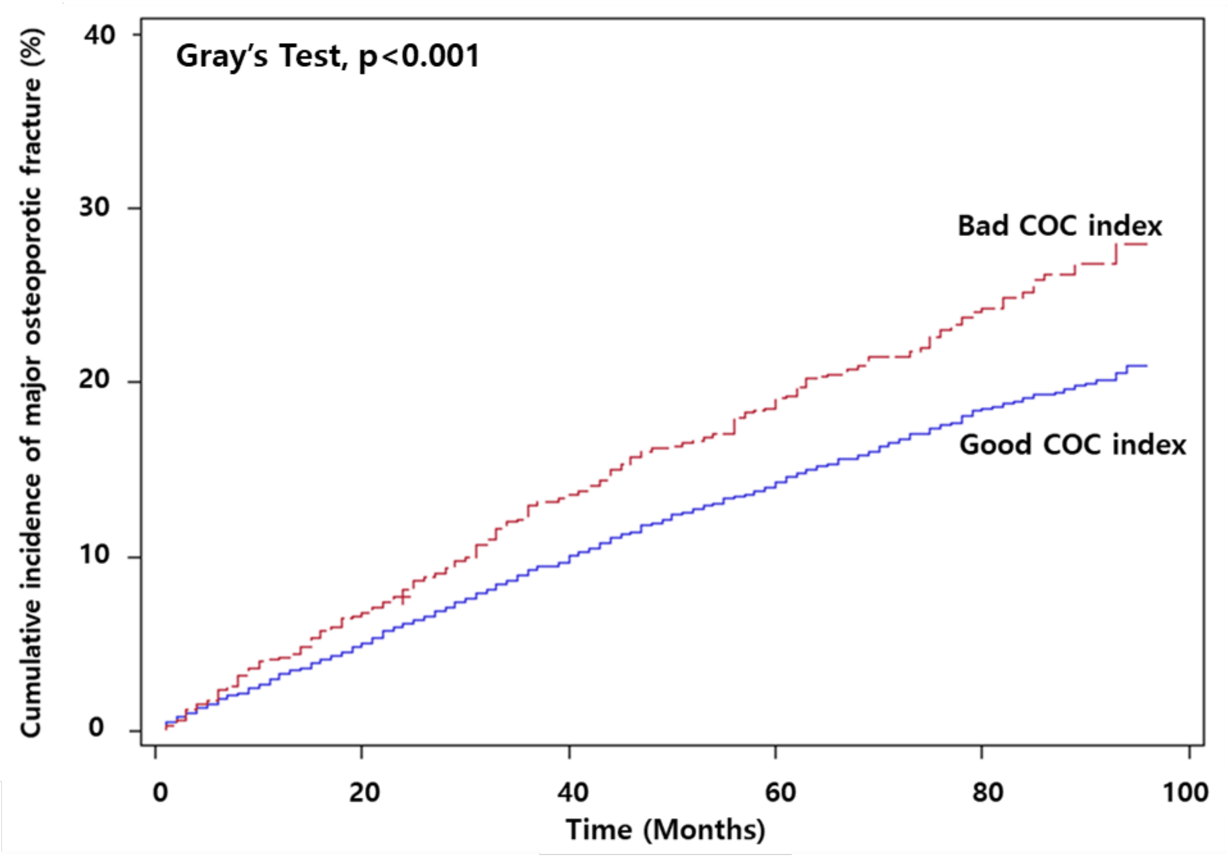

Supplement: Supplementary file 1 — Supplementary Information. [file 41598_2022_14368_MOESM1_ESM.pdf]
